# Editorial

**Published:** 1853-12

**Authors:** 


### THE MEDICAL EXAMINER.

PHILADELPHIA, DECEMBER, 1853.

### VALEDICTORY.

With this number the connection of the present editors with the Medical Examiner ceases. In terminating an editorial career which has extended through many years, and which, with one of the editors, dates from the foundation of the Journal, we beg to express our warm acknowledgements for the constant professional support that has encouraged and lightened our labors; and to tender a respectful and most cordial parting salutation to the *readers*, with whom we have so long communed. In reverting to the editorial conduct of the Journal which now passes from our control, we feel that we may at least take the credit of a steadfast and uniform endeavor to maintain in its columns the rights and honor of our Profession, and to advance the great objects of medical science.

Dr. Samuel L. Hollingsworth will assume the editorial duties of the Medical Examiner, from the commencement of the ensuing year. We have the sincerest pleasure in resigning our position in his favor, for we know no one better qualified by talents, acquirements, experience, and social and professional station, to maintain and increase the efficiency and reputation of the Journal. In its prosperity we feel the deepest interest, and our connection with it will always be among the most agreeable of our professional retrospects.

### HARVEY DEMONSTRATING THE CIRCULATION.

Though the politeness of Messrs. C. J. Price & Co., of Philadelphia, we have received a copy of a print bearing the above title, and desire to call the attention of the medical profession to it as a work of art.

The object of this work is, by furnishing a pictorial record of an incident in the life of a great man, to extend a knowledge of one of the most important discoveries in science, and to keep alive in the hearts of all who admire genius, gratitude to the memory of one of the greatest discoverers. Nothing seems more obvious, or more readily understood, than the function of the heart; but, until the time of Harvey, it was involved in the greatest obscurity, and mixed up with all manner of contradictory absurdities. And yet before his day many things were made out; the pulmonary circulation was understood, and several other essential points had been established; still the great inference had never been drawn. So often are we on the very threshold of a discovery, which by some fatality we miss; and, when it is at length made, can only express our astonishment that we were so marvellously purblind as to overlook it. The same services which Newton rendered to optics and astronomy, by his theories of light and gravitation, Harvey conferred upon anatomy and medicine by his doctrine of the circulation of the blood.

The scene represents Harvey's apartment in the palace. The king is seated in the front of the picture. The courtier who stands behind him, with his hand familiarly resting on the back of his chair, indicates the attachment and devotion of the cavaliers in those times of danger to the king. The skull and the nearly spent hour-glass behind this group may have a meaning to the moralist. The close proximity of the young prince to the philosopher indicates the gentle character of the man, and the inoffensiveness of the operation. The prince has suspended the perusal of Harvey's favorite author, for the greater excitement of his friend and tutor's demonstration. The extreme fondness for anatomical studies which in after-life characterized both Charles II. and James II. is thus explained.

The courtier behind is one of that class of gentlemen who, in reference to the advancement of social and philosophical conditions, "cares for none of these things;" he is permitting himself to be entertained by some of Harvey's opponents. These are incarnations of pedantic bigotry and stolid imbecility—the two great opponents of scientific progress—who, by insult and obloquy, show their hatred of him who dares, by asserting truth, unsettle their long-cherished absurdities.

The artist has taken great pains to preserve the likeness of Harvey, and was guided by the excellent portrait by Cornelius Jansen, in the College of Physicians, the authorities of which most kindly placed that and all the college contained concerning Harvey at his disposal.

The picture is the property of Joseph Hodgson, Esq., F. R. C. S., of Westbourne Terrace, Hyde Park, and has been reproduced in this country by Mr. G. S. Appleton, of New York.

